# Physical Activity, Loneliness, and Meaning of Friendship in Young Individuals – A Mixed-Methods Investigation Prior to and During the COVID-19 Pandemic With Three Cross-Sectional Studies

**DOI:** 10.3389/fpsyg.2021.617267

**Published:** 2021-02-02

**Authors:** Sonia Lippke, Marie Annika Fischer, Tiara Ratz

**Affiliations:** Department of Psychology & Methods, Jacobs University Bremen, Bremen, Germany

**Keywords:** relationship status, physical activity (exercise), loneliness, COVID-19, friendship (male/female), mixed-methods

## Abstract

Meaningful social interactions and regular physical activity are inversely associated with loneliness. Using a mixed-methods research design employing quantitative and qualitative research approaches, this research aimed to explore loneliness, physical activity, friendship, and experiences relating to the COVID-19 pandemic both prior to and during the pandemic. Quantitative data of (1) *n* = 363 first-year university students assessed in 2018/2019 and of (2) *n* = 175 individuals aged 18–29 years assessed in 2020 were gathered using independent self-administered online surveys. In addition, (3) *n* = 4 students were recruited for semi-structured, qualitative interviews in 2020 during the onset phase of the COVID-19 pandemic. Correlation and regression analyses as well as analyses of variance were conducted. Thematic analysis as a qualitative method was used to explore the role physical activity, friendship, and social interactions played in loneliness, particularly in times of social isolation and social distancing. Results revealed associations of varying strength between physical activity and loneliness in 2018/2019 (*r* = −0.09, *p* ≤ 0.05) and 2020 (*r* = −0.20, *p* < 0.01). In 2020, *n* = 73 (41.7%) participants felt that their loneliness had increased since the COVID-19 social and physical distancing guidelines were introduced, but this was not associated with a perceived change in physical activity (*r* = −0.05, *p* > 0.05). Analyses of qualitative data revealed three main themes: (1) the lack of deep friendships at university, (2) the positive perceived impact of team sports on feelings of loneliness, and (3) the need for real connection in times of crisis. Thus, with regard to feelings of loneliness during the pandemic, being physically active seems to be a small but potentially relevant factor among young individuals. The qualitative study suggests that first-year university students might buffer the lack of deep friendships and meaningful interactions by building social bonds in team sports. In times of physical distancing, young individuals vulnerable to loneliness may therefore require special support such as doing sports with physical distance and perceiving connected with their team for instance by digital devices and emotional coping.

## Introduction

*Perceived loneliness* denotes the feeling that one’s social needs are not being met by existing social relationships (Hawkley and Cacioppo, 2010). It is well established that this feeling is not solely related to a low quantity of social interactions. Indeed, poor social interaction quality (i.e., if individuals are lacking in meaningful interactions) is assumed to be one of the strongest predictors of loneliness (Wheeler et al., 1983; Lee and Ko, 2017). This presumption suggests that existing feelings of loneliness can be eased by generating more deep and meaningful social interactions (Hawkley and Cacioppo, 2010). Numerous studies have demonstrated the importance of examining loneliness when analyzing the impact of the Coronavirus Disease (COVID-19) pandemic (e.g., Cao et al., 2020; Hamermesh, 2020; Heidinger and Richter, 2020; Loades et al., 2020). Some studies have explicitly shown that loneliness has increased since the start of the pandemic (Elmer et al., 2020; Heidinger and Richter, 2020). Other studies have also indicated that chronically lonely individuals remained lonely, whereas those at risk for becoming lonely due to the COVID-19 pandemic consisted of specific groups such as younger individuals and those experiencing social isolation (Heidinger and Richter, 2020).

Several *critical life events* and situations, such as moving out from the parents’ house or into a different city for school, starting university, or a new job, may pose very distinct challenges to social life. Consequently, loneliness represents an important issue among young individuals and university students (Vasileiou et al., 2019) and this finding has been reported across several countries (Oezdemir and Tuncay, 2008; Diehl et al., 2018; Hysing et al., 2020). For many university students, the *transition from high school to university* co-occurs with major life changes such as leaving home and building new social relationships. This age group is associated with loneliness and transition-related changes in health behavior (Diehl et al., 2018). Furthermore, recent research suggests an increasing trend in loneliness among university students (Hysing et al., 2020). Research investigating the protective determinants of loneliness among university students is therefore required.

One such protective factor, alongside fostering meaningful friendships, is *physical activity*. Physical activity has been found to be related to loneliness (Lee and Ko, 2017). According to a systematic review by Pels and Kleinert (2016), physical activity can contribute to a decrease in feelings of loneliness. However, little is known about the mechanisms underlying this potential association (Diehl et al., 2018). Another potential protective determinant of perceived loneliness is relationship status. Research has shown that, especially in the context of a romantic partnership, *female* individuals feel lonely more often than males do (Pinquart and Sörensen, 2001). Additionally, *those without a partner or living alone* feel lonely more often than individuals in a committed relationship or those living with others (Beutel et al., 2017; Gyasi et al., 2020). Therefore, this study, using both qualitative and quantitative research methods, aims to examine the relationships between the meaning of friendship and partnership, physical activity, and loneliness in students from international universities in Europe. Moreover, physical activity can be predicted by social-cognitive variables such as intention, planning and self-efficacy (Schwarzer et al., 2008). However, rather little is known on whether such predictors also interrelate with loneliness when controlled for physical activity, and whether the association with partnership status would still be prevalent. Additionally, loneliness and physical activity have both been found to be related to work-life balance and quality of life (Fischlmayr and Kollinger, 2010; Kang et al., 2018) as well as to buffer the effects of the COVID-19 pandemic (Hu et al., 2020). Thus, the question remains as to whether physical activity and its predictors (i.e., social-cognitive variables such as intention, planning and self-efficacy) provide a meaningful addition to the explained variance of work-life balance and quality of life in regard to loneliness in general, i.e., prior to the COVID-19 pandemic.

The *COVID-19 pandemic* has already triggered a range of publications examining its effects on loneliness: Studies have demonstrated that individuals who were alone in lockdown experienced reduced happiness (Hamermesh, 2020). Among older individuals, those living alone were shown to be at higher risk for increased feelings of loneliness (Heidinger and Richter, 2020). Notwithstanding, there is other evidence suggesting that reported loneliness does not interrelate with any state orders and lockdown measures (Luchetti et al., 2020). Therefore, it is crucial to consider one’s current circumstance when examining loneliness, particularly since previous research has shown that “Compromised regulation of emotion in lonely individuals explained their diminished likelihood of performing any physical activity, and loneliness also predicted a decrease in physical activity over time” (Hawkley and Cacioppo, 2010, p. 220). Compromised emotion regulation may result from challenges attributed to COVID-19, and those maintaining physical activity might be more prone to negative effects, such as feelings of loneliness. The negative association between physical activity and loneliness might therefore become more pronounced in challenging times such as the COVID-19 pandemic.

Summarizing, in light of challenges brought along by the COVID-19 pandemic such as social distancing and increased feelings of loneliness, *friendship* and coping strategies become even more important. Therefore, this study aims to explore and evaluate various factors relating to perceived loneliness among young individuals and university students using a combination of *quantitative and qualitative research methods*. Given that no studies could be identified that used a mixed-method approach to address younger individuals during the COVID-19 pandemic, the current research aimed to fill this gap.

The following *research questions* were investigated: (1) Does physical activity relate differently to loneliness prior to and during the COVID-19 pandemic? (2) Do sex and relationship status/living situation relate to loneliness? (3a) To what extent does physical activity and its social-cognitive predictors as well as work-life balance and quality of life explain variance of loneliness in general, and (3b) To what extent does physical activity explain variance of loneliness during the COVID-19 pandemic? (4) How does the meaning of friendship relate to feelings of loneliness? and (5) How does the COVID-19 situation affect friendships and feelings of loneliness? We aim to answer research questions 1–3 using quantitative methods and data. Based on qualitative data we aim to study the research questions 1, 2, 4, and 5.

The application of a qualitative approach in addition to a quantitative one is particularly useful as it facilitates the collection of in-depth, detailed data which provide a more holistic view of the studied area (Tuffour, 2017). Quantitative research was conducted on students from an international university in Germany prior to the COVID-19 pandemic, and on a representative sample of the German population younger than 30 years of age during the COVID-19 pandemic. Qualitative research was conducted with students from universities in Europe (see Table 1).

**TABLE 1:** Characteristics of the participants in the qualitative study.

Pseudonym	Country of Origin	University	Sex	Age
Will	England	University of Sussex	Male	20
Paul	Germany	Jacobs University Bremen	Male	20
Melis	Turkey	University of Groningen	Female	20
Marielle	Honduras	University Bremen	Female	21

## Materials and Methods

### Procedure and Participants

For the *quantitative measures of this study taken prior to the COVID-19 pandemic*, we used a dataset from a first-year student sample from Jacobs University in Bremen, Germany, who were recruited using convenience sampling. The questionnaire-based survey was conducted between February 2018 and February 2019 during a lecture and was facilitated by mail-out recruitment. Students in an onboarding lecture were instructed on the purpose of the study verbally and were asked to access the survey via a link. Those who missed the class and were not in the lecture were identified using the class database and were contacted by email wherein the purpose of the study was explained. Respondents were told the purpose of the assessment was to better understand the experiences and behaviors at the university during the preceding weeks and to collect sociodemographic data. When individuals clicked on the link to the study, they received the short participant information including statements on confidentiality. Subsequently, they were asked to affirm the informed consent form before proceeding to the questionnaire. *N* = 363 (93.6%) of all 388 eligible students completed the questionnaire, provided informed consent, and were included in the analyses. Students were between 17 and 46 years of age. Further sample characteristics are reported in Table 2.

**TABLE 2:** Means and standard deviations or numbers and frequencies and correlation pattern of main study variables in 2018/2019.

		Kendall’s tau-b								
	*M* (SD) or *n* (%)	Loneliness	PA	Sex	Age	Partner status	PA intention	PA plans	PA self-efficacy	Quality of life
Loneliness in 2018/2019	2.24 (0.67)									
PA	194 (53.4%) physically active	−0.09*								
Sex	187 (51.6%) male	−0.02	0.14**							
Age	19.33 (2.27)	0.02	0.02	0.09						
Partner status	69 (18.5%) with partner	−0.10*	0.05	−0.15**	0.13**					
PA intention	2.85 (0.67)	−0.02	0.14**	0.05	−0.01	−0.02				
PA plans	2.92 (0.91)	−0.10*	0.40**	0.03	0.03	0.03	0.16**			
PA self-efficacy	3.24 (0.82)	−0.15**	0.36**	−0.01	0.03	0.10*	0.20**	0.33**		
Quality of life	3.64 (1.03)	−0.23**	0.16**	0.12*	−0.05	0.06	0.08	0.10	0.18**	
Work-life balance	2.90 (1.27)	0.16**	−0.11*	0.04	0.08	0.05	−0.03	−0.01	−0.03	−0.11*

PA, physical activity was coded: 0 = not meeting the criterion 5 days per week with 30 min or overall 2.5 h per week, 1 = meeting the criterion. Sex was coded 1 = female, 2 = male. Partner status was coded 1 = single, 2 = close relationship/married. Higher values indicate worse work-life balance. * ≤ 0.05; ** < 0.01.

For the *quantitative measures of this study taken during the COVID-19 pandemic in 2020*, a representative sample of the German population was recruited, with data of only those aged below 30 years being included in this manuscript. The questionnaire-based survey was conducted between June 08 and June 15 in the year 2020 by the company Bilendi. Using email, the company contacted individuals in their database and explained the purpose of the study. When individuals clicked on the link to the study, they received participant information which included statements on confidentiality. They were subsequently asked to affirm the informed consent form prior to continuing to the questionnaire. Experiences and behaviors during the COVID-19 pandemic and related restrictions^1^, as well as sociodemographic data were assessed. *N* = 175 individuals completed the questionnaire and were included in the analyses. Individuals were between 18 and 29 years of age. Further sample characteristics are reported in Table 3.

**TABLE 3:** Means and standard deviations or numbers and frequencies and correlation pattern of main study variables in 2020.

		Kendall’s tau-b				
	M (SD) or *n* (%)	Loneliness	Lonelier since the pandemic	PA	Sex	Living situation
Loneliness in 2020	1.95 (1.22)					
Lonelier since the pandemic	2.29 (0.93)	0.46**				
PA	81 (46.3%) more physically active	−0.20**	−0.05			
Sex	92 (52.6%) male	−0.11	−0.05	−0.01		
Living situation	137 (78.3%) with partner	−0.10	−0.13	0.04	−0.17*	
Age	24.22 (3.61)	0.05	0.02	−0.15*	0.05	−0.10

PA, physically active was coded: 0 = less active or equally active, 1 = more active. Sex was coded 1 = female, 2 = male. Living situation was coded 1 = living alone, 2 = livining together with one or more individuals. * ≤ 0.05; ** < 0.01.

For *qualitative interviews gathered in 2020*, *n* = 4 participants were recruited from four universities in Europe (*n* = 2 from Germany, *n* = 1 from the Netherlands, *n* = 1 from the United Kingdom). The recruitment was conducted via an advertisement for interviews of freshmen interested in reflecting on personal experiences with friendship and loneliness. Furthermore, interviewees would be afforded an opportunity to learn more about qualitative research methods, particularly those for evaluating the topic “Friendship and loneliness among first-year students at university.” The advertisement was disseminated to social media platform users on Facebook, Instagram, Twitter, and LinkedIn. Twenty volunteers responded to the advertisement. Only first-year university bachelor students fluent in English who were available for a 45 to 60 min Skype interview were given further information about the study, and asked to provide consent. This resulted in 5 potential study participants. One individual had a conflicting schedule, which therefore led to 4 interview participants. Thus, the participants were initially selected based on their interest in the topic. During the interview phase, the four participants first received a memo informing them about their rights to withdraw from the study and about the voluntariness of their participation before signing a consent form. Interviews were conducted via the telecommunication software Skype and took place in February/March 2020. The study received ethical approval by the Ethics Commission of the German Association of Psychology (Deutsche Gesellschaft für Psychologie, EK-A-SL022013). Dropout resulted only from structural issues such as not having sufficient time, or an unwillingness to being interviewed via the internet. The participants’ demographics are displayed in Table 1.

### Measures

The *self-administered questionnaire in 2018/2019 (pre-pandemic)* contained questions on sociodemographic characteristics, loneliness, quality of life, work-life balance, as well as physical activity, and its social-cognitive predictors.

Loneliness was assessed using the short-form version of the University of California Los Angeles Loneliness Scale (ULS-8) developed by Hays and DiMatteo (1987). The items measured the total perceived loneliness of the respondent using a four-point Likert scale ranging from “I never feel this way” (1) to “I often feel this way” (4). Questions included: (1) “I lack companionship,” (2) “There is no one I can turn to,” (3) “I am an outgoing person,” (4) “I feel left out,” (5) “I feel isolated from others,” (6) “I can find companionship when I want it,” (7) “I am unhappy being so withdrawn,” and (8) “People are around me but not with me.” Item three was reverse coded. Scoring of the ULS-8 corresponded to a mean aggregation of the eight items, with a minimum loneliness score of 1 and a maximum of 4. The scale has high internal validity (Cronbach’s Alpha = 0.84).

Physical activity was assessed with a single item worded: “Please think about your typical weeks: Do you engage in physical activity at least 5 days per week for 30 min or more (or 2.5 h during the week), in such a way that you are moderately exhausted?” which has been previously validated (Lippke et al., 2009). All individuals who answered “yes” were categorized as being “sufficiently physically active” and those answering “no” were categorized as being “physically inactive.”

Additionally, intention to perform physical activity was measured by 3 items: The stem “I have the intention to…” was combined with the three items “…perform strenuous physical activity (heart beats faster, sweating) in the future,” “… be moderately physically active (not fatiguing, mild sweating) in the future,” and “… be mildly physically active (hardly strenuous, no sweating) in the future” (Lippke et al., 2009). Planning to perform physical activity was also measured with a stem (“For the next month I already planned in detail”) combined with the three items “… which concrete physical activity I will pursue (e.g., walking),” “… where I will be physically active (e.g., in the park),” and “… on which days I will be physically active (e.g., every Tuesday),” Cronbach’s Alpha = 0.87 (Lippke et al., 2009). Self-efficacy beliefs were measured by a single item: “I feel certain that I can be physically active” adapted from Schwarzer et al. (2008). Answering options for intention, plans and self-efficacy ranged from “Completely Disagree” (1) to “Agree Completely” (4), and for intention and plans a mean score was computed.

Quality of life was measured according to Whoqol Group (1995) using the question “Please think about the last 4 weeks: How would you rate your quality of life?” Answering options were “Very poor” (1), “Poor” (2), “Neither poor nor good” (3), “Good” (4), and “Very good” (5). Work-life balance was measured with the item by Syrek et al. (2011). The statement “Please mark the option on how certain you are that you can perform each task described in the statement: I find it difficult to balance work and private life” could be rated on a scale of (1) for “Strongly disagree” to (5) for “Strongly agree,” therefore higher values indicate larger difficulties with work-life balance.

In both quantitative studies, sociodemographic information was assessed by asking participants’ sex and age. Partner status (in 2018/2019 pre-pandemic, single vs. close relationship/married) and living situation (in 2020, living alone vs. living with at least one other person) were additionally assessed.

In the 2020 assessment *during the COVID-19 pandemic*, loneliness was surveyed with the item “How often do you feel lonely?” stemming from the Center for Epidemiologic Studies Depression Scale (CES-D) developed by Radloff (1977). Adapted response options were “daily,” “multiple times per week,” “once per week,” “rarely” and “never.” Higher levels indicating greater feelings of loneliness. The change in perceived loneliness during the time of the COVID-19 pandemic was also assessed with the question “Do you feel more lonely now than before the restrictions^2^ ?” with response alternatives being “not applicable at all,” “rather not applicable,” “rather applicable” and “entirely applicable.” Higher values indicated greater feelings of loneliness since the start of the COVID-19 pandemic. Using such a single item measuring loneliness was done before (e.g., Goossens et al., 2014; Pels and Kleinert, 2016).

Increased physical activity during the pandemic was assessed using a single item “I was more active doing physical activities (e.g., running, cycling, back and abdominal exercises, online sports courses)”, with response options being “yes” or “no.”

For *qualitative interviews in 2020*, a semi-structured interview guide was designed with open-ended questions, with the interview lasting between 30 and 50 min. Questions in the interview mainly ranged from (1) How does the meaning of friendship relate to feelings of loneliness? (2) How does the COVID-19 situation affect your friendships and feelings of loneliness? (3) To what extent does physical activity relate to loneliness prior to and during the COVID-19 pandemic? and (4) How do you think relationship status and living situation relate to loneliness?

### Statistical Analysis

The association between loneliness and physical activity (*research question 1*) as well as sex, and relationship status/living situation (*research question 2*) was investigated using Kendall’s tau correlation and frequency analyses on a univariate level and linear regression on a multivariate level. *Research question 3* regarding the explained variance in loneliness was investigated by analyzing the adjusted *R*^2^ in multivariate linear regression. Additionally, *research questions 1 and 2* were investigated in more depth: To test whether there was a difference in the relationship between loneliness and physical activity between the two timepoints (*research question 1)*, we compared the correlation coefficients *post hoc* (Hemmerich, 2017). To investigate the interaction between relationship status (single vs. close relationship) or living situation (alone vs. with other individual/s) and physical activity with regard to loneliness (*research question 1 and 2*), factorial 2 × 2 ANOVAs were calculated. A further additional investigation of *research questions 1 to 3* was conducted for the assessment in 2020 by analyzing associations not only with perceived loneliness in general (static loneliness), but also with a perceived increase in loneliness compared to before the pandemic-related restrictions). All quantitative analyses were conducted using IBM SPSS 26.

### Thematic Analysis

To test *research questions 1, 2, 4, and 5*, the qualitative approach applied was based on Tuffour’s (2017) approach. Thematic analysis was chosen to analyze the data from the semi-structured interview. This method of data collection allows the researchers to have the flexibility of a guided exploration of the topic and to ask the participant to expand on what is said (Britten, 1999). The interviews were audio-recorded and transcribed. The transcriptions were then coded into keywords, which were categorized into themes.

## Results

### Quantitative Assessment in 2018/2019 Pre-pandemic

For the univariate investigation of *research questions 1 and 2*, Table 2 shows the means and standard deviations or numbers and frequencies and correlation pattern of main study variables in 2018/2019. On a correlational basis, students being physically active on a regular basis seemed to be less lonely compared to physically inactive students (*r* = −0.09, *p* ≤ 0.05). Higher levels of loneliness were significantly correlated with being single (*r* = −0.10, *p* ≤ 0.05), but not with sex (*r* = −0.02, *p* > 0.05, see Table 2 for the full correlation pattern of the main study variables).

In an ANOVA testing the interaction between physical activity and partner status, with sex and age included as covariates (*research questions 1 and 2* in more depth), only physical activity (PA) was significant with *F*_*PA*_(1,357) = 4.28, *p* = 0.04, Eta^2^ = 0.02, whereas partner status did not significantly explain variance with *F*_*Partner*_(1,357) = 1.48, *p* = 0.07, Eta^2^ = 0.01, and the interaction was not significant [*F*_PA*Partner_(1,357) = 1.86, *p* = 0.17, Eta^2^ = 0.01]. The associations between loneliness and sex and age were also not significant [*F*_*sex*_(1,357) = 0.72, *p* = 0.72, Eta^2^ < 0.01; *F*_*age*_(1,357) = 0.16, *p* = 0.69, Eta^2^ = 0.01].

**FIGURE 1:**
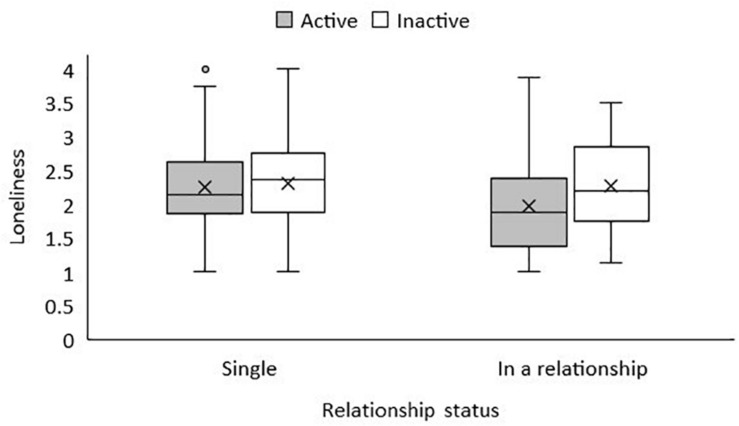
Mean values of loneliness by relationship status and physical activity in 2018/2019. The horizontal line represents the median, the X represents the mean. The boxes include the range of values from first quartile (bottom line of the box) to third quartile (top line of the box). The whiskers represent the minimum and maximum values. In case there are datapoints exceeding the ends of the whiskers, the whiskers represent the highest and lowest values which cover up to 1.5 times the interquartile range. The physical activity*relationship interaction was not significantly associated with loneliness based on 2 × 2 ANOVA.

Contrastingly, in the regression model testing whether loneliness was predicted by physical activity, sex and age on a multivariate level (*research questions 1 and 2*), the regression coefficient for physical activity was no longer significant (B = −0.03; 95% confidence interval [−0.07, 0.01]; *p* = 0.10; see model 1 in Table 1). When partner status was added, a significant association of partner status and lower levels of loneliness was revealed in model 2 [B = −0.19; 95% confidence interval (−0.37, −0.01); *p* = 0.05]. Figure 1 shows that the mean loneliness score was slightly lower in physically active students who were in a relationship compared to students who were inactive and single.

In a third step (model 3, Appendix Table 1, to test *research question 3a*), social-cognitive predictors of physical activity were included to test whether the independent relationship with partner status remained significant. The model only explained 2% of the variance in loneliness, and 4% after social-cognitive predictors were added. However, only self-efficacy [B = −0.12; 95% confidence interval (−0.21, −0.02); *p* = 0.02] was significantly associated with loneliness. When also psychological correlates of loneliness were included in model 4, the regression coefficients of both quality of life and work-life balance were significant [B_*Quality of Life*_ = −0.13; 95% confidence interval (−0.20, −0.07); *p* < 0.01; B_*Work–Life Balance*_ = 0.10; 95% confidence interval (0.05, 0.16); *p* < 0.01], explaining additional variance with an overall adjusted *R*^2^ = 0.11 (Appendix Table 1).

### Quantitative Assessment in 2020 During the COVID-19 Pandemic

In 2020, *n* = 26 (14.9%) of the assessed individuals indicated never feeling lonely, *n* = 41 (23.4%) rarely felt lonely, *n* = 38 (21.7%) felt lonely once per week, *n* = 55 (31.4%) felt lonely 2–6 days per week, and *n* = 15 (8.6%) felt lonely every day. When asked whether they felt more lonely during the COVID-19 pandemic restrictions compared to prior, *n* = 40 (22.9%) responded that this was not true at all, whereas *n* = 62 (35.4%) rated the statement as rather not true, *n* = 56 (32%) as rather true, and *n* = 17 (9.7%) agreed completely.

For the univariate investigation of *research questions 1 and 2*, Table 3 shows the correlations between the key variables assessed in 2020. A significant correlation coefficient suggested that those individuals who indicated engaging in more physical activity since the start of the pandemic scored lower on the loneliness scale compared to individuals who did not increase their physical activity during the pandemic (*r* = −0.20, *p* < 0.01). Loneliness was neither significantly correlated with sex (*r* = −0.11, *p* > 0.05), nor with living situation (*r* = −0.10, *p* > 0.05, see Table 3). There was no significant correlation of perceiving an increase in loneliness during the pandemic restrictions with reporting more physical activity (*r* = −0.05, *p* > 0.05), nor with living situation or sex.

To test explicitly whether physical activity related differently to loneliness prior to and during the COVID-19 pandemic (*research question 1* in more depth), the two cross-sectional correlations for 2018/2019 and 2020 were compared to test whether they would significantly differ. However, the Fisher’s *z* was *z* = 1.2136 with *p* = 0.225. Thus, although the two correlation coefficients appeared descriptively different, we cannot reject the hypothesis that this was observed by chance.

In an ANOVA testing the interaction between physical activity and living situation, with sex and age included as covariates (*research questions 1 and 2* in more depth), physical activity and sex were significantly associated with loneliness with *F*_*PA*_(1,169) = 6.34, *p* = 0.01, Eta^2^ = 0.04 and *F*_*sex*_(1,169) = 4.17, *p* = 0.04, Eta^2^ = 0.02. No other significant associations [*F*_*age*_(1,169) = 0.12, *p* = 0.73, Eta^2^ < 0.01; *F*_*Living*_(1,169) = 2.53, *p* = 0.11, Eta^2^ = 0.02] and no significant interaction [*F*_PA*Living_(1,169) = 0.12, *p* = 0.73, Eta^2^ < 0.01] were found.

**FIGURE 2:**
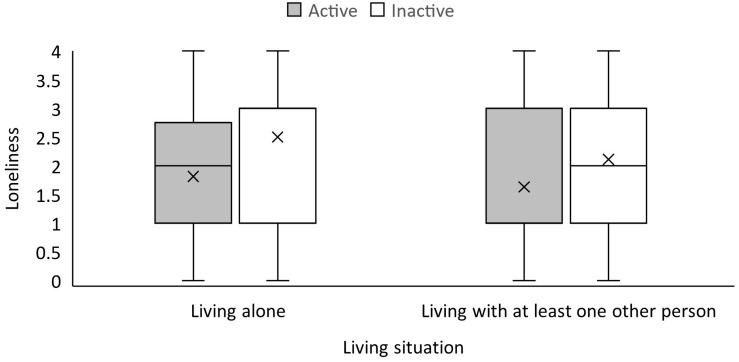
Mean values of loneliness by living situation and physical activity in 2020. The horizontal line represents the median, the X represents the mean. The boxes include the range of values from first quartile (bottom line of the box) to third quartile (top line of the box). The whiskers represent the minimum and maximum values. In case there are datapoints exceeding the ends of the whiskers, the whiskers represent the highest and lowest values which cover up to 1.5 times the interquartile range. The physical activity*living situation interaction was not significantly associated with loneliness based on 2 × 2 ANOVA.

In the regression model (testing *research question 1, 2, and 3b*) examining whether loneliness was predicted by physical activity, sex, age, and living situation, the regression coefficient for physical activity was significant [B = −0.52; 95% confidence interval (−0.88, −0.16); *p* < 0.01, see Appendix Table 2]. This finding was accompanied only by a significant association of sex and higher levels of loneliness as well [B = −0.38; 95% confidence interval (−0.74, −0.02); *p* = 0.04]. However, the model only explained 6% of the variance in loneliness. Figure 2 shows that the mean loneliness score was slightly lower in physically more active individuals who were not living alone compared to inactive individuals living alone.

In the regression model examining predictors of a perceived increase in loneliness since the start of the pandemic restrictions (Appendix Table 3, additional testing of *research question 3b*), only the static loneliness in 2020 was a significant predictor of the perceived increase in loneliness [B = 0.42; 95% confidence interval (0.32, 0.52); *p* < 0.01]. Overall, 28% of the variance in the perceived increase in loneliness could be explained (see Appendix Table 3), of which 27.7% could be attributed to the static loneliness. This indicated that the ones who felt that their loneliness had increased since the start of the pandemic-related restrictions also reported higher levels of loneliness in general compared to individuals who did not perceive their loneliness to have increased since the start of the pandemic.

### Qualitative Part in 2020 During the COVID-19 Pandemic

To better understand qualitative aspects of loneliness and friendship among students (to test *research question 1, 2, 4, and 5*), interviews were conducted and three major themes transpired: (1) the lack of deep friendship and meaningful connection at university, (2) physical activity and team environment, and (3) the need for real connection in times of crisis. The themes are illustrated by exemplary quotes listed in Table 4.

**TABLE 4:** Exemplary quote by identified theme.

Theme	Quote	Participant, line number
The lack of deep friendship and meaningful interaction at the university	“They are not people you go to. You may have 300 people you would go and have a night out with or… go to a sports tournament because you are in the team but they are not the people you would talk to when you need to talk to someone and wouldn’t talk to any of them”	1, 124–126
	“We are no longer friends we just had something in common THEN, it was so superficial”	3, 46–47
	“They probably like you for interest like if you were a good student and just with you because of that interest, or if you were good at parties”	3, 49–51
Subtheme: Supportive romantic relationships	“I have a person I can meet anytime. A person who writes to me and asks about me and worries about me”	3, 67–68
	“You have always somebody to talk to or somebody to be there with”	1, 423–423
Physical activity and team environment	“I guess your friendship in your TEAM, there will be more friction, but often it will lead you to being close”	2, 132–133
	“You would feel less lonely in comparison to, I imagine someone who just goes to lectures and seminars”	2, 143–146
	“For me personally it’s the group situation, I ONLY play team sports. You BECOME a team”	1, 187–188
The need for real connection in times of crisis	“It’s just staring at a screen, it’s not real”	4, 198–198
	“It makes certain situations more challenging because obviously people aren’t physically there to support you and the phone might just not do it”	3, 155–157
	“If you’re having a deep conversation and then you want to hug the person you cannot do it”	4, 171–172
	“I’m more…CONNECTED to a person by seeing them”	2, 160–161

#### The Lack of Deep Friendship and Meaningful Connection at University

The theme of *lacking deep friendships and meaningful connections at university* was especially prominent throughout the interviews. Most of the friendships the students stated having at university distinguished “real friends” from “university friends.” Real friends were described as being special to the student and are those with whom the student has a meaningful and deep connection. University friendships, on the other hand, were described as temporary, shallow, and not of the same quality as real friendships. All students described feeling as though many of the university friendships would not exist if they did not go to university together.

These data illustrated a hesitation among the students to confide in their university friends, which they interpreted as being due to not having meaningful interactions. Meaningful interactions were described as being the key to not feeling lonely at university. In order to prevent feelings of loneliness, the students suggested having meaningful interactions via friendships that go beyond university, as well as to form close, personal bonds. In addition, university friendships were described as existing to fulfill a specific purpose and never going beyond said purpose. For example, university friendships were deemed to only having emerged because the students shared a common goal such as succeeding academically or having a good time together.

As a sub-theme, the positive impact of being in a *supportive romantic relationshi*p emerged. Two participants indicated being in a close, romantic relationship. They described the relationships as providing a feeling of support and fostered a feeling of gratitude for having someone to turn to when feeling low. Furthermore, they mentioned being much closer to their partner than to their friends and family, and that they were able to confide everything personal to their partner.

#### Physical Activity and Team Environment

Students outlined that rather than it being the physical activity itself that makes them feel better and less lonely, underlying factors instead are involved in physical activity participation. All interviewed students agreed that there is more to participating in a sport than the exercise itself. Two main factors were deemed determinants of the positive impact of performing physical activity on feelings of loneliness: (1) the team spirit on campus, and (2) having an extroverted personality.

Being in a *team* was reported as providing a feeling of being closer to fellow students because of the shared emotions that come with winning or losing a game. It further entails getting to know team members on a more personal level that is not directly linked to the university. Most importantly, being in a team meant feeling as being a part of something as well as providing a sense of belonging, which in turn was assumed to reduce loneliness.

*Personality* was mentioned by the interviewed students as being an important factor in perceived loneliness. Also, personality was perceived as an accumulation of certain characteristics within the context of a team sport, namely being more extroverted. Essentially, the types of individual personalities that are more commonly found in team sports seem to find it easier to approach people and bond with new friends.

#### The Need for Real Connection in Times of Crisis

Given the current crisis associated with the COVID-19 pandemic, there was one theme prevalent in the data. The interviewees noticed a *need for a real connection*. This need was not perceived to be met by staying in touch via smartphone, as having a call or video chat was not considered as being meaningful. The interviewees acknowledged multiple times that staying in contact by talking on the phone did not create the same intimacy as talking face-to-face does. It was described as not enabling the same conveyance of feelings and often led conversations to feel less meaningful.

Furthermore, the fear of being geographically separated from friends and relatives was salient. Students reported wanting to physically see or experience their loved ones during times of crisis, and felt scared of not being able to do so due to travel restrictions.

## Discussion

This study used a mixed-methods approach to evaluate the associations between loneliness and factors such as the meaning of friendship, relationship status, sex, and physical activity among university students and young individuals. The quantitative data were gathered prior to and during the COVID-19 pandemic, enabling the investigation of loneliness among young individuals during “regular” times and also during the COVID-19 pandemic, which is characterized by challenges exacerbating loneliness such as physical and social distancing.

Summarizing the findings and answering the *research question 1*, namely “Does physical activity relate differently to loneliness prior to and during the COVID-19 pandemic?” the results indicate that physical activity tends to be inversely related to feelings of loneliness. The strength of this association seemed to differ between prior- and during-pandemic assessments, but a *post hoc* analysis comparing the correlation coefficients revealed that the difference was not statistically significant. The extent of the association between physical activity and loneliness was relatively small and was not found consistently in the data: After adjusting for covariates in linear regression, a significant association was only found in individuals assessed during the pandemic. This finding matches theoretical considerations that loneliness hampers physical activity or vice versa, and could suggest that those individuals maintaining their physical activity may have been able to protect themselves from negative influences related to the pandemic (Hawkley and Cacioppo, 2010). Perceived increased physical activity since the start of the pandemic, however, was not found to be related to perceived increased feelings of loneliness since the start of the pandemic-related restrictions as the only significant correlate was the general (static) loneliness. Previous studies such as a United Kingdom study found that active students were less likely to experience loneliness compared to inactive students (Budzynski-Seymour et al., 2019). The same result was also found in the general population on a meta-analytical level (Pels and Kleinert, 2016). Thus, helping young individuals to be aware of the general importance of physical activity, and to become or remain physically active even when feeling lonely may represent a potential strategy contributing to decreasing loneliness, which, however, may not be easy (Hu et al., 2020).

Nevertheless, other factors could play an important role in loneliness, which was tested with the *research question 2* “Do sex and relationship status/living situation relate to loneliness?” We found that *sex* only seems to partially interrelate with feelings of loneliness in this study, which also matched previous results (e.g., Pinquart and Sörensen, 2001). Having a *partner* was not related to loneliness pre-pandemic after inclusion of relevant psychological correlates, which is not in line with previous studies reporting such an association (Beutel et al., 2017; Gyasi et al., 2020). Just *living together* with other individuals was not related to loneliness even without controlling for relevant psychological correlates, pointing toward the importance of the relationship quality (Wheeler et al., 1983; Lee and Ko, 2017).

The findings obtained with data in 2018/2019 further shed light on the differential ability of selected determinants to predict students’ loneliness in times prior to the pandemic (*research question 3a*). The results revealed that partner status was only related to loneliness until social-cognitive predictors of physical activity, i.e., self-efficacy beliefs, were included. Self-efficacy remained a significant predictor after adding quality of life and work-life balance, and while quality of life was negatively correlated with loneliness (e.g., Kang et al., 2018), work-life balance problems were positively correlated (agreement to the item “I find it difficult to balance work and private life” was associated with a higher likelihood of reporting feelings of loneliness; Fischlmayr and Kollinger, 2010). This also might hint toward a potential mechanisms of physical activity in this association: If students have difficulties balancing their different duties and recovery from strains, physical activity can help to detach from work or studying but it could vice versa contribute to feeling more stressed by having to perform physical activity despite being busy with work (Schwarzer et al., 2008; Lippke et al., 2009). To answer the *research question 3a* “To what extent does physical activity and its predictors as well as work-life balance and quality of life explain variance of loneliness in general” we can conclude that 11% of the variance could be explained, but only if social-cognitive predictors of physical activity, quality of life and work-life balance were included. Without the latter, only 4% of the variance could be explained and without social-cognitive predictors, only 2% could be explained.

Comparing these findings to the data from 2020 during the pandemic (*research question 3b*), the model containing sex, age. living situation and physical activity explained 6% of the variance in loneliness. To answer the *research question 3b* “To what extent does physical activity explain variance of loneliness during the COVID-19 pandemic” we can conclude that the contribution of physical activity to explaining the variance in loneliness was rather small but slightly higher during the pandemic compared to before the pandemic. With regard to a perceived increase in loneliness, on the other hand, physical activity barely contributed to the 28% of explained variance in the perceived increase in loneliness, but it was mostly due to including static loneliness in the model. As social-cognitive predictors of physical activity, quality of life and work-life balance were not measured in 2020, only sex, age, physical activity and living situation could be regarded and relating to the general finding from pre-pandemic, the importance of other aspects become clear.

We were able to address the *research questions 4* “How does the meaning of friendship relate to feelings of loneliness?” and “How does the COVID-19 situation affect friendships and feelings of loneliness?” in more depth with qualitative data. The results showed that *friendships* among university students were perceived to be lacking a deeper and meaningful component. Physical activity was considered as having a protective function against loneliness due to not only its physical but also its strong social component. This finding is corroborated by studies reporting that social aspects are deemed as being important determinants of physical activity participation (Pels and Kleinert, 2016).

Furthermore, the *COVID-19 situation* seemed to greatly affect the interviewed students’ perceived loneliness, as they expressed a need for real connection rather than virtual substitutes (*research question 5*). The qualitative results further supported the quantitative findings of higher physical activity levels being associated with lower feelings of loneliness (*research question 1*). Additionally, it appears it is not only the physical activity itself that makes students feel less lonely but also other *social aspects* that are involved in participating in physical activity, such as communicating with others, having a common goal, and spending time with like-minded team players. This social aspect connects the experience of physical activity with the feeling of belonging to a team. Theories postulate this link between *attachment* and a range of mental well-being factors, such as loneliness. This may explain this association between physical activity and connectedness, i.e., loneliness (Baumeister and Leary, 1995).

The association between *relationship status* and *loneliness* was also found with the qualitative approach (*research question 2*). This pattern is not unique and has been reported in studies with adults before: For instance, Adamczyk and Segrin (2015) found that single individuals reported higher social loneliness. Thus, loneliness depends on the quality of the relationships present during interactions including during the interaction and physical activity. Regardless of whether they were in a relationship or not, deep connections appeared crucial to students’ feelings of loneliness, and were regarded as providing emotional support. Young individuals nevertheless reported a lack of those meaningful relationships at their university, and described their university friendships as shallow, temporary, and goal-driven.

## Conclusion

In conclusion, this research further highlights issues related to loneliness among young individuals in the midst of *times of change* due to university and job entrance, but also due to the COVID-19 crisis and increased digitalization in form of online-classes and media-facilitated communication. Self-isolation and social distancing might be potential reasons for perceived increases in feelings of loneliness among young individuals. With our findings we could contribute to this assumption yet not confirm it. We found that virtual communication does not appear to feel as meaningful as close physical contact does. Recent publications on the COVID-19 pandemic indeed reveal that students not having as much direct contact with family and friends as they did pre-pandemic were at a higher risk of feeling isolated (Loades et al., 2020). Individuals at increased risk for both isolation and the development of mental health problems included those who lived alone and were female, those whose integration in the student social network was weaker, and those who did not receive much social support (Elmer et al., 2020). Other research on Chinese students further highlights the impact of COVID-19 stressors on mental wellbeing as it found that COVID-19-related stressors are positively associated with anxiety symptoms among students. *Social support*, however, was negatively correlated with students’ anxiety and deemed to be a protective factor against the stressors posed by the pandemic (Cao et al., 2020). This matches our findings as it suggests a need for real, physical contact because the social environment and social support by colleagues seem to influence wellbeing and perceived loneliness.

*The strengths of this study* include the consideration of multiple relevant aspects for the investigation of loneliness in evaluative examinations. The application of a mixed-methods approach enabled a holistic and in-depth exploration of loneliness among university students and young individuals. The data were collected before and during the COVID-19 pandemic, enabling the exploration of the two very distinctive timepoints. However, even though this study was conducted at international universities, insights regarding cultural differences with respect to loneliness were not researched which clearly is a *limitation*. Another shortcoming of this study was that the data stem from cross-sectional surveys of different groups of participants and no longitudinal data were collected. Furthermore, only four participants were included in the qualitative interviews, which cannot be regarded as a representative sample. An additional weakness is that the analyzed quantitative data were gathered with different loneliness measures, which is why results cannot be simply interpreted together. Furthermore, other studies comparing a multiple indicator measure with single item assessment (such as the one in the study during the pandemic) have found “that attenuation of validity coefficients due to the shortening of the loneliness measure seems minimal, except for single-item indicators” (Goossens et al., 2014; p. 3). Thus, in further studies advanced measures instead of a single item measure should be employed whenever possible.

Also, physical activity was only assessed on a binary level, indicating whether individuals were sufficiently active according to recommendations or not in 2018/2019 and whether they were more physically active or not in 2020. A more detailed assessment might have enabled a more differentiated investigation of the association between loneliness and physical activity. Another aspect that should be further researched is the long-term effects of the COVID-19 crisis on loneliness in young individuals. The interviews were conducted during the onset of the COVID-19 crisis, and the cross-sectional nature of all three studies represents a further methodological limitation. Additionally, because recruitment for the qualitative study was conducted via social media advertisements, and interviews were performed via the internet, technologically illiterate individuals were excluded. Furthermore, selection bias could have resulted from time conflicts, interest in qualitative research methods and in the topic of friendship and loneliness. This should be taken into account when interpreting the results and when planning future studies.

Furthermore, as we explicitly selected only first year students with the quantitative study prior to 2020 and the qualitative study in 2020, the perception of friendships might not be representative for university students in general. First year students had only spent a short time at university and friendships might require more time to become meaningful. Thus, the participant selection could have affected the results as the lack of depth of university friendships can be related to this special phase of this life and friendships being relatively new. Whether this finding is generalizable to all university friendships is therefore questionable and should be tested in future studies.

*Suggestions for further research* are that it might be of great interest to longitudinally explore how perceived loneliness develops as social distancing guidelines continue over a long period, as has been done before. For instance, Luchetti et al. (2020) did not find any impact of the governmental restrictions on individuals’ loneliness in the United States. This should be validated in other countries, too. A Swiss study comparing students’ social networks and mental health before and after the lockdown recently pointed out that students’ loneliness, depressive symptoms, stress and anxiety worsened. This was attributed to stressors ranging from worries about missing out, health-related worries, to worries about the future (Elmer et al., 2020). It could also indicate that there are *vulnerable subgroups* within this crisis, and can explain why on the population level Luchetti et al. (2020) did not find any interrelations between loneliness and state orders. Such a subgroup could be, for instance, physically inactive individuals. This aspect should also be tested in the subsequent studies. Moreover, the association between physical activity and loneliness should be tested longitudinally with according data over time, also incorporating tests on psychological mechanisms and their potential buffering or causal effects.

Evidence on the importance of loneliness stemming from the COVID-19 pandemic might be used to inform the development of *policies and programs to combat the potential negative impact the COVID-19 pandemic* could have on the mental wellbeing of young citizens. This study could further be useful for university and company counselors and other staff who could benefit from insights into reasons for loneliness at schools, university, and companies. Such insight may thereby enable them to encourage the building of appropriate social environments and team sport participation as means of improving health, wellbeing, and feelings of loneliness and belonging.

To conclude, performing physical activity and engaging in meaningful social interactions seem to be relevant correlates of loneliness in young individuals but need to be further researched with appropriate measures and longitudinal study designs. There is a need to address the perceived lack of meaningful and deep friendships among peers and the demand for personal interactions. This highlights the potential negative impact of COVID-19 related restrictions such as social distancing and self-isolation on individuals’ feelings of loneliness.

## Data Availability Statement

The raw data supporting the conclusions of this article can be obtained from the first author.

## Ethics Statement

The studies involving human participants were reviewed and approved by the Ethics Commission of the German Association of Psychology (Deutsche Gesellschaft für Psychologie, EK-A-SL022013). The patients/participants provided their written informed consent to participate in this study.

## Author Contributions

SL and MF contributed to the development of this protocol and the analytical strategy as well as the overall methodology. SL collected and analyzed the quantitative data, and drafted the final manuscript. MF contributed to qualitative data collection and analysis and wrote the first draft. TR contributed to the drafting process and revising the manuscript on basis of the reviewers’ feedback. All authors contributed to this work and agreed on the submission of the final manuscript version.

## Conflict of Interest

The authors declare that the research was conducted in the absence of any commercial or financial relationships that could be construed as a potential conflict of interest.

## Footnotes

1 Restrictions during the COVID-19 pandemic included closings of schools, shops, sports clubs, swimming pools and restaurants, switching to online teaching and implementing travel restraints in addition to behavioral restrictions to reduce contacts and transmission of the virus (physical distancing included keeping a minimum distance of 1.5 m, mask wearing, prohibition of meetings exceeding more than 2 households and more than 1 person per 10 m^2^ in indoor rooms).

2 Restrictions “…span moderate recommendations such as physical distancing, up to the closures of shops and bans of gatherings and demonstrations.”

## Appendix

**APPENDIX TABLE 1:** Linear regression analysis of loneliness with 2018/2019 pre-pandemic data.

	Model 1 *R*^2^ = 0.008				Model 2 *R*^2^ = 0.019				Model 3 *R*^2^ = 0.039				Model 4 *R*^2^ = 0.114			
			95% CI				95% CI				95% CI				95% CI	
	*t*	B	LB	UB	*t*	B	LB	UB	*t*	B	LB	UB	*t*	B	LB	UB
Sex	−0.040	−0.003	−0.142	0.136	−0.376	−0.027	−0.167	0.114	−0.471	−0.034	−0.174	0.106	−0.167	−0.012	−0.149	0.126
Age	−0.107	−0.002	−0.032	0.029	0.248	0.004	−0.027	0.035	0.208	0.003	−0.027	0.034	0.114	0.002	−0.028	0.031
Physical activity	−1.674	−0.030	−0.065	0.005	−1.555	−0.027	−0.062	0.007	−0.265	−0.005	−0.046	0.035	0.395	0.008	−0.031	0.047
Partner status					−2.016	−0.186	−0.368	−0.005	−1.858	−0.171	−0.353	0.010	−1.905	−0.170	−0.346	0.005
PA intention									0.039	0.002	−0.106	0.110	0.189	0.010	−0.094	0.114
PA plans									−0.485	−0.022	−0.111	0.067	−0.575	−0.025	−0.111	0.061
PA self-efficacy									−2.401	−0.118	−0.214	−0.021	−2.138	−0.102	−0.195	−0.008
Quality of life													−4.018	−0.134	−0.200	−0.069
Work-life balance													3.891	0.104	0.051	0.156

*Linear regression results are depicted as unstandardized coefficients with corresponding 95% confidence-intervals. Adjusted *R*^2^ is reported. 95% CI, 95% confidence interval; *t*, *t*-value; B, unstandardized regression coefficient; LB, lower bound; UB, upper bound; PA physical activity.*

**APPENDIX TABLE 2:** Linear regression analysis of loneliness with data from 2020 during the COVID-19 pandemic.

	Model 1 *R*^2^ = 0.011				Model 2 *R*^2^ = 0.051				Model 3 *R*^2^ = 0.060			
			95% CI				95% CI				95% CI	
	*t*	B	LB	UB	*t*	B	LB	UB	*t*	B	LB	UB
Sex	−1.771	−0.326	−0.690	0.037	−1.819	−0.328	−0.684	0.028	−2.073	−0.377	−0.736	−0.018
Age	1.007	0.026	−0.025	0.076	0.507	0.013	−0.037	0.063	0.346	0.009	−0.041	0.059
Physical activity					−2.855	−0.523	−0.885	−0.161	−2.827	−0.516	−0.876	−0.156
Living situation									−1.660	−0.368	−0.805	0.070

*Linear regression results are depicted as unstandardized coefficients with corresponding 95% confidence−intervals. Adjusted *R*^2^ is reported. 95% CI, 95% confidence interval; *t*, *t*-value; B, unstandardized regression coefficient; LB, lower bound; UB, upper bound.*

**APPENDIX TABLE 3:** Linear regression analysis of increased loneliness with data from 2020 during the COVID-19 pandemic.

	Model 1 *R*^2^ = −0.009				Model 2 *R*^2^ = −0.013				Model 3 *R*^2^ = 0.002				Model 4 *R*^2^ = 0.281			
	*t*	B	95% CI		*t*	B	95% CI		*t*	B	95% CI				95% CI	
			LB	UB			LB	UB			LB	UB	*t*	B	LB	UB
Sex	−0.552	−0.078	−0.357	0.201	−0.553	−0.078	−0.358	0.201	−0.856	−0.122	−0.403	0.159	0.290	0.035	−0.206	0.277
Age	0.348	0.007	−0.032	0.046	0.231	0.005	−0.035	0.044	0.048	0.001	−0.038	0.040	−0.160	−0.003	−0.036	0.031
Physical activity					−0.628	−0.090	−0.374	0.193	−0.586	−0.084	−0.366	0.198	1.060	0.131	−0.113	0.376
Living situation									−1.885	−0.327	−0.669	0.015	−1.169	−0.173	−0.466	0.119
Static loneliness													8.189	0.417	0.317	0.518

*Linear regression results are depicted as unstandardized coefficients with corresponding 95% confidence-intervals. Adjusted *R*^2^ is reported. 95% CI, 95% confidence interval; *t*, *t*-value; B, unstandardized regression coefficient; LB, lower bound; UB, upper bound.*
